# The prediction, diagnosis and management of complications in monochorionic twin pregnancies: the OMMIT (Optimal Management of Monochorionic Twins) study

**DOI:** 10.1186/s12884-017-1335-3

**Published:** 2017-05-26

**Authors:** Fiona L. Mackie, R. Katie Morris, Mark D. Kilby

**Affiliations:** 1 Centre of Women’s and Newborn’s Health & Institute of Metabolism and Systems Research, College of Medical and Dental Sciences, University of Birmingham, Edgbaston, Birmingham, B15 2TT UK; 2 Fetal Medicine Centre, Birmingham Women’s NHS Foundation Trust, Edgbaston, Birmingham, B15 2TG UK

**Keywords:** Monochorionic, Twin-twin transfusion syndrome, Biomarker, Prediction, Fetoscopic laser ablation

## Abstract

**Background:**

Monochorionic twin pregnancies are at increased risk of complications due to sharing a single placenta and potentially developing unbalanced vascular anastomoses. Complications include twin-twin transfusion syndrome (TTTS) which affects 10–15% monochorionic twins, and if untreated has a 70–90% perinatal loss rate. We are currently unable to predict which twins will develop complications or to what severity. We have previously shown differences in angiogenic and placental growth factors in maternal blood in pregnancies complicated by TTTS compared to twin pregnancies not complicated by TTTS but matched for gestation. There is also evidence to suggest that abnormal ultrasound measurements recorded in the first trimester (nuchal translucency and crown-rump length) may be associated with severe TTTS later in pregnancy, however the detection rate is only reported as 52%. We hypothesize that if these changes precede the development of the clinical syndrome, this may increase the sensitivity and specificity of detecting adverse pregnancy outcomes.

**Methods:**

This cohort study has retrospective and prospective elements. In the retrospective cohort we will measure factors (decided based on preliminary work and a systematic review and meta-analysis) in stored maternal blood samples taken in the first-trimester, extract first-trimester ultrasound measurements and match these to pregnancy outcome. The prospective cohort will be divided into a “screening” cohort and “complicated” cohort. The screening cohort will undergo serial maternal blood sampling at 12, 16 and 20 weeks; we will extract ultrasound measurements and match to pregnancy outcome. The complicated cohort will comprise of women referred to the Fetal Medicine Centre with complications of monochorionicity. If the decision is taken to undergo fetoscopic laser ablation we will take maternal blood samples and amniotic fluid samples pre- and post-laser treatment. The same factors will be measured in the prospective cohort as informed by the retrospective study.

**Discussion:**

This study aims to increase knowledge surrounding the pathology of complications in monochorionic twins, to aid future diagnosis and management.

**Trial registration:**

ISRCTN 13114861 (retrospectively registered)

## Background

All twin pregnancies have increased risk over singletons, with monochorionic (MC) twins demonstrating the highest risk. Thirty per cent of twins are MC, but they experience 75% of all twin complications, the pathologies of which are closely linked to the fact that they share a single placenta and thus can develop unbalanced vascular anastomoses [1]. Complications that are specific to monochorionicity include twin-twin transfusion syndrome (TTTS), selective intrauterine growth restriction (sIUGR) and twin anaemia polycythaemia sequence (TAPS) [2]. TTTS complicates 10–15% of MC twins and usually occurs between 15 and 26 weeks. At present, the prognosis is dependent upon early treatment and method of treatment: if untreated it is associated with a perinatal loss rate of 70–90%. A staging system, the Quintero system, exists but correlates poorly with outcome [3]. Even with apparently ‘successful’ antenatal treatment (with fetoscopic laser ablation (FLA)), TTTS is still associated with 10% neurological morbidity. We are currently unable to accurately predict which twins will develop complications or how severe they will be. There is some evidence that first trimester ultrasound screening of MC twin pregnancies between 11 and 13^+6^ weeks may predict adverse pregnancy outcome. These ultrasound features include: a) discordance in fetal nuchal translucency thickness (NT) with an increase in the recipient fetus [4, 5] and b) discordance in fetal crown rump length (CRL) measurements between the MC twins [5]. Although ultrasonographic features have a reasonable sensitivity and specificity in terms of allocating risk of severe TTTS, such screening is still associated with a significant false positive and false negative rate as the detection rate is only 52% [6].

We have recently reported significant differences in maternal serum between women with twin pregnancies complicated by TTTS, and twin pregnancies not complicated by TTTS but matched for gestational age [7]. These differences were seen in alpha-fetoprotein (αFP), β-hCG, plasma vascular endothelial growth factor (VEGF)-C, angiopoietin (Ang)-2 levels and the ratio of soluble vascular endothelial growth factor receptor-1 (sVEGFR-1) (also known as soluble fms-like tyrosine kinase-1. [sFlt-1]) to placental growth factor (PlGF) levels. We hypothesize that if these changes precede the development of the clinical syndrome, this may increase the sensitivity and specificity of detecting adverse pregnancy outcomes. The objective of this paper is to describe the aims and study design of our ongoing platform of work exploring complications in MC twin pregnancies.

## Methods

### Aims of study

The aim of our research is to improve knowledge of complications in MC twin pregnancies, and incorporate this knowledge into a predictive model in the future. Specifically we will investigate:

1. If abnormal first-trimester ultrasound measurements (NT and CRL) are associated with the development of complications later in pregnancy.
2. The effects that these complications and their treatment have on:
  - angiogenic factors in maternal serum/plasma
  - placental growth factors in maternal serum/plasma
  - angiogenic factors in amniotic fluid
  - placental growth factors in amniotic fluid

### Study design

This cohort study has both retrospective and prospective elements. There will be 1 retrospectively recruited first-trimester cohort, 1 prospectively recruited “screening” cohort, and 1 prospectively recruited “complicated” cohort. In all aspects of the work monochorionicity must have been confirmed using the ‘T’ sign on first-trimester ultrasound scan.

#### Retrospective cohort

We will initially perform a retrospective study of stored maternal serum microbiology samples and Down Syndrome screening samples taken in the first trimester from MC twin pregnancies as part of routine booking at Birmingham Women’s NHS Foundation Trust. These samples are routinely stored for 2 years in the microbiology and clinical chemistry department, and equate to approximately 170 samples. We will measure maternal angiogenic factors and placental growth factors in these samples, collect first trimester ultrasound measurements (CRL and NT) and patient characteristics and pregnancy outcome data. As Down Syndrome screening samples are received from 31 sites in the UK, we will ask clinicians at these sites to assist in data collection using a pre-designed proforma. Where clinicians feel unable to help with data collection, we will visit the sites and collect the outcomes ourselves. The primary outcome will be whether participants developed a complication in pregnancy (TTTS, TAPS, sIUGR, single or double intrauterine demise) and the severity of these complications. Secondary outcomes will include delivery (gestation at delivery, mode of delivery, birth weight, condition of twins at delivery) and neonatal outcomes (admission to neonatal unit, diagnosis of comorbidity, particularly neurological). The maternal angiogenic factors and placental growth factors measured will be decided based on our preliminary work (see above) and a systematic review and individual patient data (IPD) meta-analysis (see PROSPERO CRD42015024975 for full protocol). As this is a retrospective study and the results of our study will not affect patient care, patients whose blood samples we are using in this retrospective study will not be consented or informed of the results. However, all women under the care of Birmingham Women’s NHS Foundation Trust are aware that the hospital is research active, and that their blood samples may be used in this way, and are given the option of opting out if they so wish. Samples from patients who have indicated that they do not want their samples to be used for research will be excluded from this study. This retrospective study will inform the subsequent prospective study.

#### Prospective cohort

**Fig. 1:**
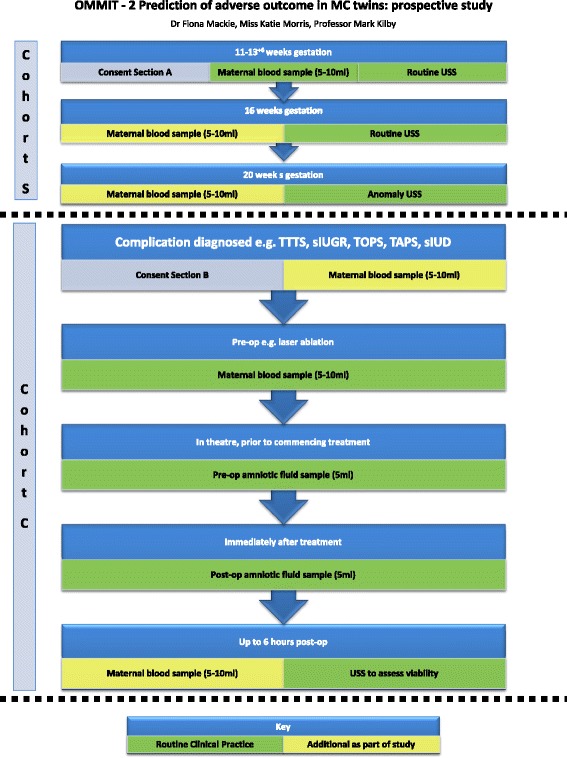
OMMIT-2 Prediction of adverse outcome in MC twins: prospective study

The prospective study will run over 2 years (August 2015–August 2017) and there will be 2 cohorts: a “screening” MC twin cohort (Cohort S), and a cohort who have developed complications (Cohort C) (see Fig. 1). In both cohorts, informed written consent will be obtained. The “screening” cohort (Cohort S) will be approached in the ‘multiples’ antenatal clinic where all twin pregnancies are managed at Birmingham Women’s NHS Foundation Trust. We will ask participants with confirmed MC twin pregnancies to consent to 2 extra bottles of blood being taken during their routine Down Syndrome blood test or booking blood tests, and for permission to record their first trimester ultrasound measurements, and access the above outcome data as specified in the retrospective study. We will then repeat the blood sampling at 16 weeks and 20 weeks gestation when they attend for routine ultrasound scans. If the participant does not develop any complications, this will be the end of her involvement in the study and we will just collect the outcome data. However, if she develops a complication (e.g. TTTS, TAPS, sIUGR, single intrauterine demise) she will be invited and consented to enter the second prospective “complicated” cohort.

Women who develop complications are referred to the West Midlands Fetal Medicine Centre. The second cohort (Cohort C) will have serial maternal blood tests performed (pre-FLA and 6 h post-FLA) looking at the biomarkers informed by the IPD meta-analysis and retrospective study. A small amniotic fluid sample will be collected pre-FLA when the port for the fetoscope has been inserted into the recipient sac in the uterus, and a second small sample taken post-FLA when the ablation has been completed. The recipient twin will have polyhydramnios as it is part of the diagnostic criteria for TTTS, and it is routine clinical practice to perform amnioreduction at this point. All samples (serum and amniotic fluid) will be spun down, aliquoted, frozen and stored at−80° Celsius. We will assess the same factors in the amniotic fluid as in the maternal serum. Data will be collected on the ultrasound scans performed as part of routine clinical management of these conditions, as will pregnancy outcome data. Quintero staging will be used to classify TTTS [8].

### Expected number of participants

In the retrospective study cohort, these samples are routinely stored for 2 years and equate to approximately 170 samples in total.

Regarding the prospective cohort S: Birmingham Women’s NHS Foundation Trust has 7,900 deliveries per annum, approximately 2% of all maternities are multiple pregnancies and thus would equate to 158 maternities per annum. Of these, approximately 30% are MC twins (47 MC twins per annum) and 70% of women with an MC pregnancy will consent to Down syndrome screening (33 MC twins per annum). If we propose that 5 per year will be lost by miscarriage or decline participation in the study, then we predict recruitment of approximately 28 MC twin pregnancies per annum, which equates to 56 maternities over 2 years of proposed recruitment.

At the West Midlands Fetal Medicine Centre we treat approximately 40–60 women a year with TTTS, which equates to 80–120 women over 2 years. If we allow for a 50% recruitment rate and incomplete follow-up, results in 40–60 sets of parents participating over 2 years in cohort C.

### Data management and data analyses

At recruitment, each participant will receive a unique study identifier to allow linkage of data. Data will be collected using a purposely designed proforma (as per the retrospective cohort) and manually input onto a secure electronic database. Hard-copies of data will be kept in a locked filing cabinet, in a swipe-card access department at Birmingham Women’s NHS Foundation Trust. We will ensure that we remain compliant with the Trust’s Information Governance policies at all times.

Descriptive data will be reported. First trimester ultrasound measurements (NT and CRL) and serial serum biomarkers will be compared between participants in cohort S who go on to develop complications, and participants in cohort S who do not develop complications. The serum biomarker results from the 16 and 20 week blood samples from participants in cohort S will also act as the control group for cohort C participants. Additionally serum and amniotic fluid biomarkers will be compared longitudinally (i.e. pre- and post-FLA) for each participant in cohort C, to evaluate the potential effect of FLA on the biomarkers. The appropriate statistical tests will be used. Chi-squared will be used for categorical variables, and student’s t-test (paired and unpaired where appropriate) for continuous variables. Logistic regression models will be created to assess the association between variables and outcomes and presented as odds ratios (OR) with a 95% confidence interval (CI). Different cut-offs will be used for TTTS (Quintero staging) and sIUGR (EFW <10^th^ centile, <5^th^ centile).

## Discussion

This study aims to increase knowledge surrounding the pathology of complications in MC twins, which in turn is hoped to aid future diagnosis and management. To our knowledge, this is the first study to look at these serum and amniotic fluid biomarkers in such a large cohort of MC twin pregnancies.

Although we are one of the largest Fetal Medicine Centres in the UK, the number of participants eligible for inclusion is insufficient to power the development of a prediction model, and may cause false negative results. The results of this study may inform a larger, multi-centre study to be conducted over several years so as to create a prediction model to triage MC twin pregnancies as high-risk or low-risk for complications, and thus requiring more or less surveillance antenatally.

There will be some women who will have booked at Birmingham Women’s NHS Foundation Trust and will be recruited to both the first-trimester screening cohort and the “complicated” cohort. Parents are routinely informed of the potential complications of MC pregnancy at booking, and by seeing these women in the first-trimester, this will give us the opportunity to inform them of the second part of the prospective study and they will have at least 4 weeks to consider participation should a complication develop. However, as a tertiary Fetal Medicine Centre we receive a lot of referrals from around the UK for second opinions and to perform treatment for MC twin complications, e.g. FLA. These referred patients will also be eligible for inclusion in the “complicated” cohort, although we will not have first-trimester blood samples for them. Unfortunately, due to the rapid progression of complications, these parents may have a limited time to consider participation. However, this is still in-keeping with ethical approval.

Although we will be unable to gain consent for participants in the retrospective study, we intend to publish our results on the TAMBA website to which the public have access. We also hope to present them at international conferences and publish in leading medical journals.

We expect to lose some participants to follow-up, and to be unable to collect outcome data for some patients who may have moved areas, or have had their baby(ies) transferred out to different neonatal units.

## Conclusions

This study hopes to increase knowledge of the pathological processes involved in the continuum of MC twin complications, and allow us to predict which MC twins will develop complications so that we are better able to target surveillance and therapy, and ultimately improve outcomes for these families.

## Abbreviations

Ang-2: Plasma angiopoietin
CI: Confidence interval
Cohort C: “Complicated” cohort
Cohort S: “Screening” cohort
CRL: Crown rump length
EFW: Estimated fetal weight
FLA: Fetoscopic laser ablation
IPD: Individual patient data
MC: Monochorionic
NT: Nuchal translucency
OR: Odds ratio
PAPPA: Pregnancy associated plasma protein A
PlGF: Placental growth factor
sFLT-1: Soluble fms-like tyrosine kinase-1
sIUGR: Selective intrauterine growth restriction
sVEGFR-1: Soluble vascular endothelial growth factor receptor-1
TAPS: Twin anaemia polycythaemia sequence
TTTS: Twin-twin transfusion syndrome
VEGF-C: Plasma vascular endothelial growth factor
αFP: Alpha feto-protein
β-hCG: Beta human chorionic gonadotrophin
